# Reconstruction of intestinal microecology of type 2 diabetes by fecal microbiota transplantation: Why and how

**DOI:** 10.17305/bjbms.2021.6323

**Published:** 2021-11-09

**Authors:** Kaijian Hou, Shuo Zhang, Zezhen Wu, Dan Zhu, Fengwu Chen, Zi-Ning Lei, Weiting Liu, Chuanxing Xiao, Zhe-Sheng Chen

**Affiliations:** 1 Department of Endocrine and Metabolic Diseases, Longhu Hospital, The First Affiliated Hospital of Medical College of Shantou University, Shantou, Guangdong, China; 2 Department of Endocrine and Metabolic Diseases, Shantou University Medical College, Shantou University, Shantou, Guangdong, China; 3 Department of Pharmaceutical Sciences, College of Pharmacy and Health Sciences, St. John’s University, Queens, New York, USA; 4 Department of Teaching and Research Section, College of Nursing, Anhui University of Chinese Medicine, Hefei, Anhui, China; 5 Department of Pharmacy, College of Traditional Chinese Medicine, Fujian University of Traditional Chinese Medicine, Fuzhou, Fujian, China

**Keywords:** Fecal transplant, intestinal microecology, type 2 diabetes

## Abstract

Type 2 diabetes (T2D) is a chronic metabolic disease characterized by hyperglycemia due to insulin resistance. Mounting evidence has correlated T2D to alterations in the composition of gut microbiota. Accordingly, targeting the gut microbiota has become an emerging strategy for T2D management. The aim of this article is to get a better insight into the rationale for targeting gut microbiota in T2D treatment. Thus, we herein reviewed the change of gut microbiota composition in T2D, factors shaping gut microbiota, and potential mechanisms behind the contribution of gut microbiota to T2D pathogenesis. At present, it has become possible to use intestinal microorganism capsules, bacteria liquid, and other preparations to carry out fecal microbiota transplantation for the treatment and intervention of T2D with insulin resistance and immune-mediated type 1 diabetes.

## INTRODUCTION

The level of sugar (glucose) in human blood is under strict control by a hormone known as insulin [1]. However, when the control mechanism is impaired, hyperglycemia can occur. This is the basis for the development of diabetes. Normal human blood sugar level ranges from 70 mg/dL to 99 mg/dL, whereas diabetic patients will have a fasting blood sugar level higher than 126 mg/dL [2]. The most well-known symptoms of diabetes include, but are not limited to, frequent urination, excessive thirst, increased hunger, and other unexplained body changes. Without ongoing management, excess sugar in the blood will lead to many complications, such as neuropathy, nephropathy, retinopathy, and cardiovascular disease [3-6]. As per the World Health Organization report, there are about 442 million people worldwide who are suffering from diabetes, over 90% of whom have type 2 diabetes (T2D) (https://www.who.int/health-topics/diabetes). T2D is mainly characterized by insulin resistance, which means the human body cannot use insulin effectively [7]. High-fat dietary habit and sedentary lifestyle are known as major factors contributing to T2D progression, while the underlying mechanisms are still not fully understood.

Gut microbiota is a collective term for microorganisms, including bacteria, fungi, archaea, and viruses, in the gastrointestinal tracts [8]. Even though the gut microbiota is composed of various microorganisms, the bacteria components have received the most research interest. With the advancement of next generation sequencing technology, it has become possible for researchers to comprehensively evaluate bacterial diversity and detect the abundance of microbes in certain environments. In the human gut, the number of bacteria has been estimated to be over a hundred trillion [9]. All bacteria in the human gut are classified into over 1000 species, whereas about 99% of them come from 30 to 40 species [10]. It is noteworthy to state that the abundance of each species is not distributed equally. The most dominant bacterial phyla in the human gut are *Firmicutes*, *Bacteroidetes*, *Actinobacteria*, and *Proteobacteria*. Most bacteria belong to the genera *Bacteroides*, *Clostridium*, *Faecalibacterium*, *Eubacterium*, *Ruminococcus*, *Peptococcus*, *Peptostreptococcus*, and *Bifidobacterium* [11].

The microbiota can be considered a special “organ” that plays a significant role in human health [12]. Like other organs, loss of homeostasis in the gut microbiota can lead to different diseases including allergies, Celiac’s disease, gastric cancer, and obesity [13,14]. In the past two decades, accumulating evidence has associated gut microbiota with T2D as well [15]. Inspired by this knowledge, targeting gut microbiota has been proposed as a promising strategy for T2D treatment. To get a better insight into the rationale behind this strategy, we herein review the factors shaping gut microbiota, changes of gut microbiota composition in patients with T2D, and potential mechanisms behind gut microbiota contribution to T2D pathogenesis. In addition, we also discuss the advancements in targeting gut microbiota for T2D treatment.

## FACTORS SHAPING GUT MICROBIOTA

**FIGURE 1:**
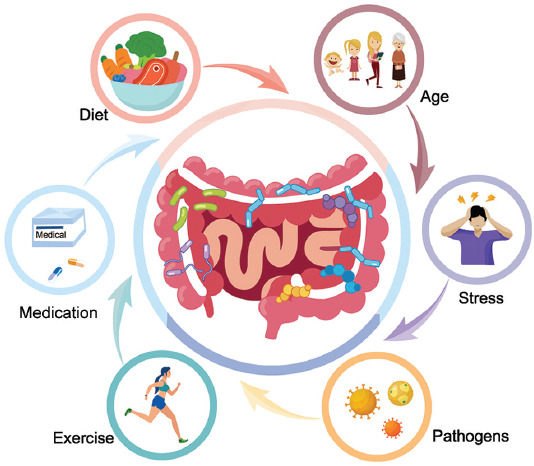
Factors affecting the structure of gut microbiota. Various factors contribute to alterations in gut microbial communities, including diet, exercise, consumption of medicine (especially antibiotics), aging, stress, and infection by pathogens.

The gut microbiota is generally stable over time, but the composition can be altered due to external perturbations [16]. By far, people have identified various factors contributing to alterations in gut microbial communities, such as delivery pattern, diet, medication, and exercise (Figure 1). The human gut microbiota is established via contact with the environment during early years of life [17]. Starting as early as during vaginal delivery, newborns can acquire microbes habituating in the mother’s vaginal canal [18]. Indeed, several studies have revealed a strong correlation between the microbiota in the digestive tract of newborns and the microbial communities in the vaginal canal of their mothers [19]. On the other hand, infants born by cesarean section (C-section) mainly acquire the first colonization of bacteria from the hospital environment and skin of the mother [20,21]. Now it is known that the gut bacteria diversity in infants born by C-section is lower than vaginally delivered ones, but the delivery pattern-related differences in gut microbiota mostly subside after the 1^st^ year [20]. Of note, although most vaginal and skin bacteria will not take hold in the gut of the infant, their presence may differentially affect the colonization abilities of other bacteria. For example, infants born by C-section are less likely to be colonized by *Bifidobacterium and Bacteroides* in the 1^st^ week after birth [22]. Furthermore, *Escherichia, Shigella, and Bacteroides* are underrepresented in infants born by C-section [23]. These differences may explain why babies born by C-section have relatively higher risk of chronic immune disorders [24].

Food consumption is the key way through which humans acquire bacteria into the gut. It is now well established that diet plays a pivotal role in shaping the gut microbiota [25]. Studies in mice and humans have shown that short-term shift in dietary macronutrients could alter the gut microbiome rapidly and reproducibly [26,27]. But these bacterial changes caused by short-term dietary interventions are usually transient. Moreover, the short-term alterations only occur among a limited number of bacterial taxa; the core bacterial taxa are resilient to most temporary outside influences [28]. Unlike the limited and transient alterations of gut microbiota caused by short-term dietary changes, long-term dietary habits can result in broad and consistent shifts of gut bacteria [29]. Common types of diets can be broadly classified into two categories: Animal-based diets containing high fat/low fiber and plant-based diets containing low fat/high fiber. In humans, consumption of a diet composed entirely of animal products resulted in enrichment of bile-tolerant bacteria and depletion of bacteria that metabolize plant polysaccharides [26]. Similarly, mice studies have also associated high fat/low fiber intake with an increase in *Firmicutes* and decrease in *Bacteroidetes* in gut bacteria composition [30].

In addition to diets, taking medicines, especially antibiotics, can dramatically change the amount and type of bacteria in the gut as well. Antibiotics are intentionally administered for the depletion of pathogenic bacteria, but commensal gut microbes could also be killed or inhibited due to the broad-spectrum activities of antibiotics [31]. Different antibiotics have different antimicrobial spectra and will result in different changes in the gut microbiome. For example, vancomycin can decrease fecal microbial diversity and the number of gram-positive bacteria, particularly the *Firmicutes* phylum [32], whereas amoxicillin does not change total bacterial numbers and microbial diversity significantly [33]. Usually, the changes of gut microbiota caused by antibiotics will recover to pretreatment conditions within days or weeks after cessation of antibiotic treatment. However, for some specific gut bacteria, the effects of certain antibiotics can last for years or forever. This will cause a permanent change in the gut microbial community [34]. Besides antibiotics, non-antibiotic medicine can also modulate the gut microbiota composition. A large Dutch-Belgian population study showed that drugs (including osmotic laxatives, progesterone, TNF-α inhibitors, and rupatadine) had large influence on microbiota composition [35]. Other studies have shown major effects of commonly prescribed proton pump inhibitors on the microbial community, which probably could explain why people taking these drugs have higher rates of gastrointestinal infection [36]. Recently, several human and animal studies emphasized that metformin, a drug commonly used for T2D treatment, altered the gut microbiota composition as well [37]. It enhances the growth of mucin-degrading *Akkermansia muciniphila* and several short-chain fatty acid (SCFA)-producing microbiota in the gut of T2D patients, which benefits them in maintaining glucose metabolism homeostasis [38].

The earliest evidence on the effects of exercise on the gut microbiota was derived from observations of Matsumoto et al. who reported that, in rats, running resulted in a variation in the gut microbiota composition [39]. Evans et al. further demonstrated that exercise could prevent obesity and induce changes in the percentage of major bacterial phyla in mice. Interestingly, they also found a negative correlation between the distance ran with the *Bacteroidetes*-*Firmicutes* ratio [40]. In humans, a major study conducted within professional rugby players demonstrated that exercise enriched the diversity of gut microflora, which is consistent with the animal studies. Clarke et al. also explored exercise for its impact on the gut microbiota of professional athletes from an international rugby union squad. They found a higher alpha diversity of gut microorganisms representing more than twenty distinct phyla in athletes compared with controls [41]. In addition, Bai et al. demonstrated that daily exercise increased gut microbial diversity with a *Firmicutes* enrichment microbiota in young children and adolescents [42]. By analyzing the fecal microbiota of individuals with different fitness levels, Estaki et al. found that exercise was correlated with increased gut microbial diversity with enrichment in butyrate-producing taxa, resulting in increased butyrate production, which is an indicator of gut health [43]. Taken together, these studies provide evidence for a beneficial impact of exercise on gut microbiota and human health.

Symbiosis and codependency are universal among different subsets of gut microbiota. Physiologically, the microbiota maintains a homeostatic state [44]. The metabolite product of some bacteria may provide necessary nutrients to other species. For example, the *Bifidobacterium adolescentis* strain is able to degrade fructo-oligosaccharides and starch to produce lactate and acetate. Butyrate-producing bacteria cannot directly utilize fructo-oligosaccharides and starch, but can utilize lactate and acetate as growth substrates. Therefore, *B. adolescentis* can facilitate the proliferation and expansion of butyrate-producing species by cross-feeding. This cross-feeding-dependent symbiotic relationship is also found in *Rhodopseudomonas palustris, Escherichia coli, Methanobrevibacter smithii, Bacteroides thetaiotaomicron, Eubacterium rectale*, and *hetaiotaomicron* [45]. In contrast, some metabolites accumulated in the gut may be toxic to other microbes, and microbial biotransformation of these toxic metabolites may be restricted to specific species. One of the most striking examples is conjugated bile acids, which can inhibit the growth of bacteria in the duodenum and jejunum [46]. Deconjugation by *Lactobacilli, Bifidobacteria, Clostridium*, and *Bacteroides* is the key step in reducing the toxicity of bile acid [47]. Deconjugated bile acids can be further used by bacteria or reabsorbed by the liver for bile acid enterohepatic circulation [48]. Thus, the loss of specific populations of microbiota may lead to the alteration of metabolites and the microenvironment in the gut, which, in turn, affect the growth of other members of the gut microbiota.

Other factors affecting the composition of gut microbial profiles include exposure to pathogens, age, and psychological stress. Colonization of pathogenic bacteria can induce inflammation in the gastrointestinal tract, which destabilizes the gut microbial community and its function. By another way, the toxins produced by the pathogens can also affect the survival of certain commensal bacteria [49]. The changes to the gut microbiota with age are mainly results of weakened immune systems, less physical activity, and dysfunction of the digestive tract or malnourishment in older people [50]. As for psychological stress, studies have shown that it can affect gut motility, visceral perception, and intestinal permeability [51]. These effects on the gastrointestinal tract can negatively affect the composition of gut microbiota.

## ALTERATION OF GUT MICROBIOTA COMPOSITION IN T2D

Although gut microbiota is generally stable since the early years of life, alterations in gut microbiota composition have been frequently noted in various diseases [13,44]. These alterations are also frequently reported in T2D patients. While the reports were not always consistent, they all demonstrated the correlation between alteration in gut microbiota composition and T2D. In a human study, Larsen et al. found a significant decrease in the abundance of *Firmicutes* in T2D patients. They also found the ratio of *Bacteroidetes* to *Firmicutes* to be positively correlated with the plasma glucose concentration [52]. Consistent with their report, Ridaura et al. demonstrated that *Bacteroidetes* could mediate the degradation of branched-chain amino acids (BCAA), and the BCAA may potentiate insulin resistance [53]. However, several other studies have indicated different observations. In a metagenomic study, Qin et al. observed a decrease in *Bacteroidetes* and an increase in *Firmicutes* in T2D [54]. Furthermore, Turnbaugh et al. found that a lower, but not higher, ratio of *Bacteroidetes* to *Firmicutes* was correlated with obesity and insulin resistance [55]. The reason for these conflicts is still not clear and thus needs further investigations.

At the genus level, negative associations between *Bifidobacterium*, *Bacteroides*, *Faecalibacterium*, *Akkermansia*, and *Roseburia* and T2D were commonly reported. In a human study, Wu et al. observed a significant decrease of *Bifidobacterium* in T2D when compared with healthy participants [56]. Their conclusions were strengthened by a couple of animal studies demonstrating the role of *Bifidobacterium* in improving glucose tolerance [57,58]. Similarly, administrations of *Bacteroides acidifaciens* and *Bacteroides* *uniformis* also ameliorated glucose intolerance and insulin resistance in diabetic mice, which implied possible beneficial roles of *Bacteroides* also in human health [59,60]. Consistent with the findings of decreased butyrate-producing bacteria in T2D, a lower abundance of *Roseburia* and *Faecalibacterium* in T2D has been reported in multiple studies as well [61]. As a member of the *Akkermansia*, *A. muciniphila* was documented to mediate the negative effects of IFN-gamma on glucose metabolism [62], which implicated a negative association between the abundance of this bacterium and T2D.

Compared to the number of negatively associated bacteria, there are fewer studies reporting increase of specific gut microbes in T2D. In a study with 217 Mexican subjects, Chávez-Carbajal et al. found a higher abundance of *Comamonadaceae* in pre-diabetes and *Sutterella* in T2D [63]. Interestingly, Remely et al. demonstrated that *Akkermansia* was more highly abundant in T2D. This is contradictory to a previous study that indicated a negative association between *Akkermansia* and T2D. The reason for this contradiction still needs further investigation. In addition to *Akkermansia*, they also found a higher abundance of *Enterobacteria* in T2D. Other bacteria that appear in higher abundance in T2D include *Ruminococcus*, *Fusobacterium*, and *Blautia* [64].

To summarize, the general rationale for reshaping gut microbiota in T2D treatment is supported by the notion that microbiota composition is altered in this disease, which can inform the treatment strategy. However, these findings of changes in gut microbiota in T2D patients are not always consistent. This makes it challenging to propose a standard protocol for treating T2D by targeting the gut microbiota. Moreover, it is still controversial whether the change of gut microbiota is the reason or result of T2D progression. For more accurate and convincing conclusions, broader and deeper investigations are still necessary.

## ROLE OF GUT MICROBIOTA IN T2D PATHOGENESIS

**FIGURE 2:**
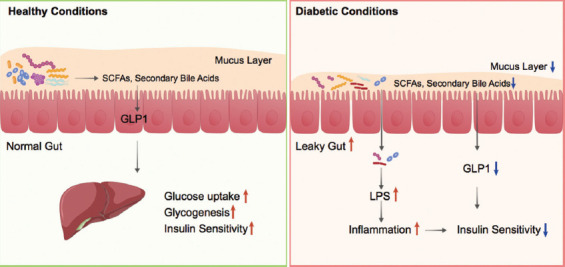
Roles of gut microbes in T2D pathogenesis. Gut microbiota plays pivotal roles in maintaining gut homeostasis and glucose metabolism (left). Dysbiosis in gut microbiota is thought to be involved in the pathogenesis of T2D by different mechanisms (right). SCFAs: Short chain fatty acids; LPS: Lipopolysaccharide; GLP1: Glucagon-like peptide-1.

Gut microbiota can interact with various host sensing and signaling pathways, leading to a modulation of the endocrine system, immune system, nervous system activity, and hence, the predisposition to metabolic diseases such as T2D [65-67]. Multiple mechanisms behind the contribution of gut microbiota to T2D have been illustrated, such as induction of low-grade inflammation, perturbation of energy homeostasis, and glucose metabolism (Figure 2). Herein, we summarize those mechanisms to review how changes in gut microbiota can contribute to T2D pathogenesis.

In general, T2D is a chronic metabolic disease characterized by insulin resistance [68]. Low-grade chronic inflammation is one of the most important pathophysiological factors resulting in the progression of insulin resistance in T2D [69]. Lipopolysaccharides (LPSs), derived from the outer layer of Gram-negative bacteria membrane, play important roles in promoting the secretion of pro-inflammatory cytokines. As a pathogen-associated molecular pattern, circulating LPS can bind to the Toll-like receptors to stimulate the innate immune system and trigger a local or systemic inflammatory response [70]. High-fat diet (HFD) has been associated with increased levels of circulating LPS. In an animal study, Cani et al. found that a 4-week HFD led to a 2~3-fold increase in the concentration of plasma LPS, which could be the result of elevated LPS in gut microbiota. They also found that continuous infusion of LPS in mice triggered a whole-body inflammatory response and insulin resistance in the liver, which suggested the role of LPS signaling in the development of T2D [71]. Accordingly, less circulating LPS and insulin resistance were developed in high fat-fed germ-free mice and mice receiving antibiotics [72].

Increasing levels of circulating LPS could be caused by the uptake of LPS in chylomicrons secreted from intestinal epithelial cells or increased intestinal permeability. Some evidence showed that a thinner gut mucus layer is often observed in T2D [73]. The mucus layer in the gastrointestinal tract is an efficient system for protecting the epithelial cells from bacterial infection [74]. The structure of the mucus layer is affected by the gut microbiota. On the one hand, the presence of gut microbiota induces expression of the genes encoding mucin 2 and galactoside 2-alpha-L-fucosyltransferase 2, thereby affecting mucus strength and mucin glycan structure. On the other hand, gut microbiota can use mucus components as their energy source when the main source of nutrients, plant polysaccharides, is lacking [75]. Among a synthetic community consisting of 13 human-derived gut microbial species that cover the dominant phyla in the human gut, *A. muciniphila* and *Barnesiella intestinihominis* have been found to be exclusively fed on mucin O-glycans. Therefore, the increase in *A. muciniphila* and *B. intestinihominis* in the gut of T2D patients could be responsible for their thinner gut mucus layer than normal cohorts [76]. One consequence of a thinner gut mucus layer is a leaky gut with enhanced permeability. In this condition, LPS will get through the gut barrier and lead to low-grade chronic inflammation [77].

One significant beneficial contribution of gut microbiota to humans is their ability to degrade dietary fiber and starch. The metabolites generated by gut microbiota have a pivotal role in the maintenance of homeostasis of host physiology [78]. As the major product of dietary fiber fermentation by gut microbes, SCFAs, namely acetate, propionate, and butyrate, have been shown to exert multiple beneficial effects on mammalian energy metabolism [79]. In non-obese diabetic mice, SCFAs were able to protect them against insulitis and slow the progression of diabetes [80]. It is known that SCFAs participate in regulating the metabolism of both fatty acids and glucose that are closely related to obesity and T2D. Acetate and propionate are used as substrates for gluconeogenesis and lipogenesis in the liver, whereas butyrate is an important energy substrate for colonic mucosa cells [81]. Moreover, SCFAs can bind to the G protein-coupled receptors on the surface of enteroendocrine L-cells to promote the production of glucagon-like peptide-1 (GLP1), which leads to improved insulin sensitivity [82]. In T2D, decreased levels of fecal SCFAs were common, which may result from less dietary fiber consumption and a decreased number of SCFA-generating bacteria [83].

Besides the SCFAs, changes in microbiota may also correlate with T2D progression due to resulting changes in the metabolism of bile acids. Primarily produced in the liver, bile acids are circulated to the gut where the gut bacteria can metabolize them into secondary bile acids [84]. Secondary bile acids, in turn, bind to the G protein-coupled receptor TGR5 on enteroendocrine L-cells to promote the production of GLP1 and insulin sensitivity. Dysbiosis of gut flora may profoundly affect the bile acid pool in T2D [85]. An increase in primary bile acids and a decrease in secondary bile acids were observed in serum of T2D patients, which was thought to be the result of decreased *Bacteroidetes*/*Firmicutes* ratio [86].

Collectively, the role of gut microbiota in the pathogenesis of T2D is complex. The current evidence indicates that the change in gut microbiota may cause intestinal epithelial leakage and absorption of macromolecules from the intestine, which, in turn, triggers a systemic immune response and low-grade inflammation, changes lipid and glucose metabolism, eventually leads to insulin resistance and T2D.

## TARGETING GUT MICROBIOTA IN T2D TREATMENT

Gut microbiota modulation is a clinically promising strategy to treat diseases related to imbalances in gut microbiota. The alteration of gut flora composition in T2D supports the rationale for targeting gut microbiota in its treatment. Methods for modulating the gut flora balance include the use of prebiotics, probiotics, and fecal microbiota transplantation (FMT). Here, we will provide a brief overview of the advancements and challenges associated with the methods for modulating gut flora in T2D.

Prebiotics are non-digestible food ingredients that benefit the host by affecting the activity or composition of gut microbiota. Usually, prebiotics are composed of oligosaccharides or short polysaccharides such as galacto-oligosaccharides, inulin, galactofructose, and oligofructose. One common characteristic of all prebiotics is that they cannot be degraded by the digestive enzymes in the intestine. Thus, they can reach the colon to be consumed by certain gut bacteria like *Lactobacilli* and *Bifidobacteria* [87,88]. Regardless of considerable animal studies suggesting the beneficial roles of prebiotics for T2D cases, evidence in humans is limited. In a recent systematic review of 27 human studies, Colantonio et al. found that 20 studies demonstrated a beneficial effect of prebiotics on metabolic and inflammatory markers, with 19 of them showing improved glycemia [89]. Of note, all studies investigated different prebiotics and all participants were females. Broader studies with more participants and with both genders are needed. Moreover, effects may vary depending on prebiotic type and patient characteristics. Therefore, personalized prebiotics supplementation could be a trending strategy.

Probiotics, often termed “good bacteria,” are live microorganisms that benefit the host’s health by inhibiting pathogenic bacteria growth, producing SCFAs, and stimulating the immune system. While the Food and Drug Administration has not approved probiotics to treat diseases, many studies show the beneficial role of probiotics for T2D patients. In a meta-analysis, probiotic administration was shown to reduce fasting blood glucose and HbA1c in T2D patients [90]. However, other studies found that probiotics have no clear benefits to T2D management [91]. The inconsistent results may be due to differing types and doses of probiotics. In addition, there are also some doubts about whether enough bacteria can survive the stomach acids and reach the gut. To become established in the gut, the probiotic strains also must outcompete the resident microbiota and occupy an ecological niche. Because the resident microbiota is inherently resistant to outside colonizers, the probiotic strains may fail to colonize and function in the gut. Therefore, further investigation is needed to better determine the probiotic dose and duration of administration that may be effective for T2D management.

FMT is the procedure of transplanting fecal bacteria from healthy donors to restore the community and function of gut microbiota in patients. Compared to prebiotics and probiotics treatment, FMT treatment directly delivers fecal bacteria to the colon and to restore microbiota. The therapeutic benefit of FMT was well established in the treatment of *Clostridium difficile* infections (CDI). It can resolve 80-90% of infections caused by recurrent *C. difficile* that does not respond to antibiotics. In guidelines for the treatment of recurrent CDI, FMT is now considered standard of care [92]. Inspired by the efficacy of FMT in CDI, investigators explored the benefit of FMT in T2D treatment. In an animal study, Wang et al. found that rebuilding the microbiota in T2D mice by FMT could reverse insulin resistance [93]. Accordingly, clinical trials in humans also demonstrated that FMT helped alleviate insulin resistance in T2D patients. Vrieze et al. confirmed that the transfer of gut microbiota from lean donors increased insulin sensitivity in individuals with metabolic syndrome [94]. Meanwhile, Kootte et al. also found that lean donor FMT in obese metabolic syndrome patients improved insulin sensitivity [95]. Interestingly, they observed that the beneficial effects of lean donor FMT were transient, which indicates that recurrent FMT might be necessary. Despite the promising results revolving around FMT in T2D, some concerns about the non-desirable side effect of the FMT remain. For example, Harsch and Konturek reported that a patient with chronic radiation colitis developed adhesion ileus 2 days after FMT [96]. The transmission of communicable diseases and increased infection risk of FMT also need to be addressed.

## DISCUSSION

There are a large number of microbes in the human intestines, forming an extremely complex microecosystem, and maintaining a relatively balanced state to sustain the normal physiological functions of the body. In recent years, the participation of intestinal microbiota has also been increasingly recognized in the occurrence and development of T2D [97]. Compared with individuals with high fecal bacterial population, those with low fecal microbial population were more likely to have obesity, metabolic disorders, and inflammatory phenotypes [98]. Intestinal flora can regulate T2D through multiple targets. The specific mechanisms of action are categorized as follows.

Firstly, intestinal flora can affect T2D through regulation of bacterial metabolites. In recent years, studies have found that bacterial metabolites play an important role in flora-mediated sugar metabolism. Studies have shown that imidazole propionate, a histidine-derived metabolite produced by microorganisms, may be a bridge connecting intestinal flora to T2D. Elevated imidazole propionate level in the blood has been reported in T2D patients, and may be the cause of impairment of glucose tolerance and insulin signaling [99]. HFD can drive changes in bile acids and inflammatory signal transduction, insulin resistance and glucose metabolism. This can be mitigated by antibiotics, which can alter intestinal microbiota [100]. SCFA is an important regulator of intestinal microbiota and is involved in various physiological functions, such as maintaining an acidic environment, providing energy, and maintaining the integrity of the intestinal mucosal barrier. Therefore, SCFA is increasingly being regarded as a nutritional target for the prevention and treatment of T2D.

Second, intestinal flora can affect bile acid metabolism. Bile acids are the final products of cholesterol catabolism, and they are also new signaling molecules that act as metabolic regulators [101]. Recent studies have revealed that bile acid receptors (BARs) like nuclear farnesoid X receptor and cytoplasmic G protein-coupled receptor 5 (TGR5, M-BAR) are involved in regulating gluconeogenesis, peripheral insulin sensitivity, glycogen synthesis, as well as GLP1 and insulin secretion [102]. The important regulatory functions of bile acids in glucose metabolism make it a potential target for diabetes treatment. The TGR5 signaling pathway is essential in regulating intestinal GLP1 secretion *in vivo*, and pharmacological targeting of TGR5 may constitute a promising incretin-based strategy for the treatment of diabetes and related metabolic disorders [103]. Thus, maintaining the balance of the intestinal flora is key to equilibrating bile acid metabolism, and therefore critical to improving T2D.

Another major mechanism by which intestinal flora affects T2D is the metabolism of BCAAs. BCAAs include three essential amino acids: Leucine, isoleucine, and valine. They are important nutritional metabolism signal molecules in the human body, and BCAA synthesis is closely related to the state of the intestinal flora [104]. BCAAs and aromatic amino acids (AAAs) are associated with insulin resistance and development of diabetes [105,106]. Increasing evidence shows that the changes in the composition of the gut microbiota can modulate BCAA metabolism, and thus contribute to the development of diabetes. For example, many bacterial species are involved in BCAA synthesis and AAA decomposition. Studies have shown that BCAAs promote the uptake of glucose in the liver and skeletal muscle [107] and enhance glycogen synthesis through the phosphatidylinositol 3-kinase or protein kinase C pathway [106]. In animal models, reducing dietary BCAAs increases insulin sensitivity; there is also a negative correlation between dietary BCAA intake and T2D risk [108].

Finally, intestinal flora can affect T2D by regulating LPS-mediated inflammation. Chronic inflammation is a key characteristic of T2D. Various pro-inflammatory factors contribute to the pathogenesis of T2D [69]. Disorders of intestinal flora will produce a large amount of LPS [109], which subsequently results in many varying biological activities. Li et al. found that the two types of lactobacilli, G15 and Q14, could significantly reduce the number of Gram-negative bacteria, lower the levels of LPS and inflammatory factors, and alleviate T2D [110].

Intestinal dysbiosis has been identified as a potential factor contributing to the growing prevalence of diabetes [111]. As alluded to earlier, FMT helps rebuild new intestinal flora and may prevent intestinal diseases. A recent double-blind, randomized clinical trial showed that FMT enhanced the composition of fecal microbiota in obese T2D patients, and combination with lifestyle modification led to a more favorable pattern of FMT-mediated compositional shift in microbiota, such as enrichment in probiotic *Bifidobacterium spp*. and *Lactobacillus spp*. [112]. Another double-blind, randomized clinical trial for obese patients with metabolic dysfunction demonstrated that the patient groups receiving oral FMT exhibited a significant shift in the beta diversity of the microbiota compared to those getting placebo FMT [113]. In this study, the authors further found that the patients receiving FMT supplemented with low-fermentable fiber, compared to the other FMT group receiving high-fermentable fiber supplement, had a greater degree of change in the composition of fecal microbiota, particularly an increase in the species related to insulin sensitivity improvements, such as *Phascolarctobacterium*, *Bacteroides steroids*, and *Bacteroides caccae*. This demonstrates the response of adaptive host microorganisms to therapeutic intervention and may indicate a possible benefit of FMT combined with dietary fiber [113]. To make targeting gut microbiota safer and more effective in T2D patients, more clinical studies are still needed. Of note, most ongoing gut microbiota studies are still focusing on bacterial members but neglecting the potential roles of viruses and fungi in the complex gut communities. Investigation on non-bacterial microorganisms in the gut may give us more integrated knowledge of the relationship between gut microbiota and our health.

## CONCLUSION

By reviewing the change of gut microbiota composition in T2D, factors shaping gut microbiota, and potential mechanisms behind the effect that gut microbiota has on T2D pathogenesis, we provided a better insight into the rationale for targeting gut microbiota in T2D treatment. Accordingly, despite some challenges, targeting gut microbiota emerges as a promising strategy for T2D treatment.
